# PPAR and Liver Injury in HIV-Infected Patients

**DOI:** 10.1155/2009/906167

**Published:** 2009-04-15

**Authors:** Maud Lemoine, Jacqueline Capeau, Lawrence Serfaty

**Affiliations:** ^1^INSERM, UMR_S U893, CDR Saint-Antoine, 75012 Paris, France; ^2^UPMC University Paris 06, UMR_S 893, CDR Saint-Antoine, 75012 Paris, France; ^3^APHP Tenon and Saint-Antoine Hospitals, 75012 Paris, France; ^4^Service d'Hépatologie, Hôpital Saint-Antoine, AP-HP, 184 rue du Faubourg Saint-Antoine, 75571 Paris Cedex 12, France

## Abstract

Due to the introduction of active HIV antiretroviral treatment, AIDS-related morbidity and mortality have markedly decreased and liver diseases are now a major cause of morbidity and mortality in HIV-infected patients. Chronic liver injury encompasses a wide spectrum of diseases due to HCV and HBV coinfection, drug-related toxicity, and NASH. HIV-infected patients who are receiving treatment present with a high prevalence of metabolic complications and lipodystrophy. Those patients are at high risk of nonalcoholic fatty liver disease, the liver feature of the metabolic syndrome. This review will focus on (1) the liver injuries in HIV-infected patients; (2) both the current experimental and human data regarding PPAR and liver diseases; (3) the interactions between HIV and PPAR; (4) the potential use of PPAR agonists for the management of HIV-related liver diseases.

## 1. Introduction 

The efficacy of highly active antiretroviral treatment (HAART) has resulted in a considerable improvement in the life expectancy of HIV-infected patients. As a consequence, liver diseases have emerged as a key issue in the management of HIV-infected patients, and they are now a major cause of morbidity and mortality [1]. Chronic liver injuries in HIV-infected patients encompasses a wide spectrum of liver diseases mostly consisting of coinfection with hepatitis B and C viruses, excessive alcohol consumption, drug-related toxicity, and more recently identified fatty liver disease. Over the last few years, a set of metabolic alterations has also emerged in HIV-infected patients treated with HAART, including the lipodystrophy syndrome, which is closely associated with insulin resistance. The syndrome typically associates visceral fat hypertrophy, limb lipoatrophy, dyslipidemia, and insulin-resistance, thereby resembling a caricature of the metabolic syndrome. Those patients are at high risk of nonalcoholic fatty liver disease, the liver feature of the metabolic syndrome.

Peroxisome proliferator-activated receptors (PPAR) constitute a family of nuclear receptors, some of them being expressed in the liver. They are involved in glucose and lipid metabolism, in insulin sensitivity [2] and in many other physiological processes, particularly inflammation fibrogenesis [3] and carcinogenesis. Experimental and human data have portrayed PPAR as potential molecular players in chronic liver diseases. Interactions between some HIV proteins and PPAR reinforce the potential role of these nuclear receptors in the development of HIV-associated liver injuries. In addition, the development of new nonhepatotoxic ligands made it possible to use PPAR agonists as new therapeutic targets in liver diseases. For these reasons, studies in the field of PPAR are of high concern, not only to basic researchers but also to clinicians for the management of liver diseases. This review will focus on (1) the liver injuries in HIV-infected patients; (2) both the current experimental and human data regarding PPAR and liver diseases; (3) the interactions between HIV and PPAR; (4) the potential use of PPAR agonists for the management of HIV-related liver diseases.

## 2. Liver Injuries in HIV-Infected Patients: Which Disease Has Which Prevalence?

### 2.1. Coinfection with Hepatitis B and C

Due to their common routes of transmission, chronic hepatitis B and C viruses are often associated with HIV infection and are present in 10% and 30% of HIV-infected patients, respectively.

It has been clearly established that HIV infection significantly changes the natural history of HBV and HCV by enhancing their viremia levels [4]. HIV also worsens the histological course of HBV and HCV by increasing the severity of fibrosis and accelerating the risk of cirrhosis and hepatocellular carcinoma [5, 6].

### 2.2. Excessive Alcohol Consumption and Drug-Related Toxicity

Excessive alcohol consumption has been observed in one-third of HIV-infected patients [5, 7]. In addition, alcoholic hepatitis is more frequent and more severe, suggesting a specific sensitivity to alcohol in HIV-infected patients [8].

All classes of antiretroviral drug have been associated with liver toxicity. Drug-related toxicity is more frequent in patients treated with some nonnucleoside reverse transcriptase inhibitors (NNRTIs) or protease inhibitors (PIs) and in patients with preexisting liver injuries due to alcohol, HBV, or HCV [9]. Some nucleoside reverse transcriptase inhibitors (NRTIs) are able to induce microvesicular steatosis with lactic acidosis, potentially leading to hepatic failure [10].

### 2.3. Nonalcoholic Fatty Liver Disease: An Underestimated Cause of Liver Injury in HIV-Monoinfected Patients

Steatosis, the main feature of fatty liver, is defined as abnormal fat accumulation in hepatocytes related to metabolic abnormalities and insulin resistance, toxic injuries (alcohol, drugs), or viral infections, in particular with HCV. Nonalcoholic fatty liver disease (NAFLD,) also called metabolic liver disease, has become the most common cause of chronic liver injury in HIV-uninfected patients [11], with an estimated prevalence in the general population ranging from 14% to 31% [12–14]. One-third of those patients have histologic signs of fibrosis and necroinflammation, indicating the presence of nonalcoholic steatohepatitis (NASH) [11]. Such liver injuries are likely to lead to cirrhosis, liver, failure and hepatocellular carcinoma. Insulin resistance plays a central role in the development of liver steatosis, but the precise molecular mechanisms leading to steatohepatitis and fibrosis remain undefined.

Lipodystrophy is a frequent long-term side effect of antiretroviral therapy, being reported in 40 to 50% of HIV-infected patients receiving HAART [15]. As a result of insulin resistance and/or visceral fat hypertrophy, HIV patients with HAART-related lipodystrophy are considered at risk of NAFLD. In addition, NAFLD has been recently demonstrated to be an early marker of cardiovascular disease, which has also become an emergent issue in HIV-treated patients with HAART over the last decade [16]. These data underscore the importance of assessing the presence of NAFLD in HIV-infected patients with antiretroviral treatment who are particularly at risk of metabolic disorders.

In HIV-monoinfected patients with HAART-related lipodystrophy, few data are available on the true incidence of NAFLD and most of the studies used indirect tools for the diagnosis of steatosis, whereas the benchmark is histologic assessment of liver fat content through the use of biopsy [13]. Indirect evidence of fatty liver has been suggested in HIV patients with lipodystrophy by demonstration of a significant correlation between alanine aminotransferase (ALT) serum levels and insulin resistance [17]. However, it is thought that liver injury is poorly correlated with liver enzyme serum levels in patients with NAFLD [18]. By using proton spectroscopy, Sutinen et al. found increased liver fat related to the severity of insulin resistance in 25 HIV patients with lipodystrophy [19]. Moreno-Torres et al. found intrahepatic triglycerides deposits in 17 of 29 HAART recipients, 4 of whom (13.8%) had liver fat contents compatible with the diagnosis of liver steatosis [20]. More recently, Hadigan et al. identified hepatic steatosis in 42% of their patients [21]. Mohammed et al. demonstrated that HIV-infected patients with NAFLD had lower body mass indices than HIV-seronegative patients [22], suggesting that NAFLD may be associated with factors other than those classically observed in obesity, including direct HIV infection and antiretroviral therapy. Among 225 HIV-infected patients enrolled in a recent study, 83 (36.9%) were diagnosed with NAFLD using tomodensitometry [23]. Two studies used liver biopsy for the diagnosis of unexplained chronic transaminase elevation and NASH was observed in over half of HIV-infected patients in the absence of other causes of chronic liver diseases, with a close association with insulin resistance [24, 25]. Guaraldi et al. [23] showed that NAFLD was associated with lipodystrophy and waist size, suggesting a relationship between adipose tissue, insulin resistance, and fatty liver. In light of these studies, NAFLD may be considered a common liver disease in HIV-monoinfected patients. HAART-related insulin resistance and lipodystrophy likely play a major etiologic role but their mechanisms remain to be determined. Some PPARs are expressed in the liver, the central organ in the balance of glucose and lipid metabolism, and as a result they could be involved in metabolic-related liver injuries.

### 2.4. PPAR and Liver Injury

PPAR are transcription factors belonging to the family of nuclear receptors [26]. When activated by ligand binding, PPAR are able to activate promoters and to modulate the expression of target genes. They exist in three isoforms, PPAR*α*, PPAR*β*/*δ*, and PPAR*γ*. This review will focus on PPAR*α* and PPAR*γ*, as the functions of PPAR*β*/*δ* in glucose and lipid metabolism are less established.

PPAR*α* is highly expressed in the liver, and its functions are better documented than those of PPAR*γ* which are synthesized at lower levels. PPAR*α* is the most abundant nuclear receptor in the liver and is mainly expressed by the hepatocytes [27] though also by the stellate cells, biliary cells, endothelial cells, and Kupffer cells [3, 28–30].

These nuclear receptors are involved in glucose and lipid metabolism and also in nonmetabolic functions including inflammation, tissue repair, cell proliferation, differentiation, carcinogenesis, and fibrosis [3, 31, 32]. Several studies mostly conducted in vitro and in animal models have suggested that these nuclear receptors could be involved in the development of liver injuries including steatosis, inflammatory injuries, and fibrosis. There is very little data available on PPAR expression and functions in HIV-infected livers. As no data have been published in HIV/HCV or HIV/HBV coinfected patients, this review will focus on HIV-monoinfected patients, particularly those with metabolic disorders.

### 2.5. PPAR and Steatosis

Steatosis is closely associated with insulin resistance and impairment of glucose and lipid homeostasis. Activation of PPAR*α* directly regulates genes involved in fatty acid uptake by increasing the expression of the fatty acid transport protein (FATP) and the fatty acid translocase (FAT) [33]. PPAR*α* also promotes fatty acid oxidation in the peroxisomes, as well as in the mitochondria, reducing the fatty acid pool available to the liver for triglyceride synthesis [34]. In addition, PPAR*α* activation decreases triglyceride levels by enhancing lipoprotein lipase (LPL) expression [35] and by inhibiting apolipoprotein C-III in the liver [36]. Activators of PPAR*α* include diverse chemicals such as endogenous molecules (fatty acids/steroids) and xenobiotics (fibrate lipid-lowering drugs) [37–39].

The potential role of PPAR*α* in the development of liver steatosis is mostly based on experimental data conducted in murine lacking PPAR*α*. PPAR*α*−/− mice display obesity and serious liver steatosis without excessive food intake and PPAR*α* activation by fibrates reverses insulin resistance and reduces weight [40]. There is a little data available in the human liver on PPAR*α* liver expression and its role in liver diseases. In chronic hepatitis C with steatosis, reduced levels of PPAR*α* mRNA have been demonstrated [41]. In HIV-infected patients with HAART-related lipodystrophy and NAFLD, Lemoine et al. did not observe changes in the liver expression of PPAR*α* mRNA compared to NASH patients without HIV and to normal liver controls [24]. No additional data have been published on human liver disease to date.

PPAR*γ* is a transcription factor that regulates the gene expression involved in lipid metabolism and in adipocyte differentiation [42]. PPAR*γ* is highly expressed in the adipose tissue under two isoforms (PPAR*γ*1 and PPAR*γ*2) that are generated by the same gene through altering splicing [43]. Whereas the functions of PPAR*γ* are well established in the adipose tissue, they remain hypothetical in the liver.

PPAR*γ* activation plays a role in various physiological and pathological events, including adipocyte differentiation, insulin sensitivity and regulation of lipid metabolism. The natural ligands of PPAR*γ* remain unknown. There is evidence that small lipophilic compounds, such as polyunsaturated fatty acids and fatty acid derivatives (eiocosanoids), bind and activate PPAR*γ* [44]. Thiazolinediones are synthetic ligands used as antidiabetic drugs, as they have been reported to enhance insulin sensitivity [45]. In animal as well as in human livers, PPAR*γ* levels are much lower than those of PPAR*α*. Several murine models of obesity and diabetes, including ob/ob, A-ZIP, aP2/DTA, and KKAy have been shown to develop fatty livers that express high levels of liver PPAR*γ* mRNA [46, 47]. In addition, mice lacking PPAR*α*−/−, which develop liver steatosis, also expressed high hepatic PPAR*γ* mRNA [40]. These results suggest a potential etiologic role of PPAR*γ* in fatty liver disease. Few studies have been conducted in human livers. PPAR*γ* is expressed at low levels and its expression is decreased in HCV-monoinfected patients [41] and HIV-infected patients [24].

### 2.6. PPAR and Inflammation

Many experimental data are in favor of anti-inflammatory activities of PPAR. PPAR*α* and PPAR*γ* have been shown to downregulate inflammatory response genes by inhibiting the STAT, AP-1, and NF-*κ*B transcriptional pathways in human hepatocytes and monocytes [31, 48, 49]. In primary human hepatocytes, Delerive et al. suggested the anti-inflammatory effects of fibrates, which are PPAR*α* agonists, by increasing I*κ*B expression and antagonizing NF-*κ*B activation [49]. By regulating antioxidant enzyme activities, such as catalase, PPAR*α* agonists may also reduce the oxidative stress [50].

These anti-inflammatory effects have also been validated in vivo. Acute hepatitis induced in PPAR*α*−/− or in PPAR*γ*+/− animals is particularly severe with exacerbation of liver inflammatory injury [51, 52]. One of the suggested mechanisms is the ability of PPAR*α* activation to inhibit the nuclear translocation of NF-*κ*B [49]. In livers of PPAR*α* wild-type mice, treatment with fibrates resulted in I*κ*B induction, whereas no increase was observed in PPAR*α* null mice [49].

Not only in alcoholic models of rats and mice but also in hepatocytes treated with ethanol and acetaldehyde, Lee et al. recently showed a downregulation of PPAR*α* mRNA [53]. In humans treated with fenofibrate, a decreased level of blood cytokines (TNF*α*, IL-6, IFN*γ*) was found [54]. No relationship has been demonstrated between the extent of PPAR expression and liver inflammation in human livers.

### 2.7. PPAR and Liver Fibrosis

PPAR*γ* has been shown to be expressed in quiescent hepatic stellate cells (HSCs) and its expression and activity are decreased during HSC activation in rats in vitro and in vivo [3, 28, 55]. The treatment of rat HSC with PPAR*γ* ligands prevents their activation in vitro. In addition, the in vivo treatment of rats with synthetic PPAR*γ* agonists prevents fibrosis induced by toxic or bile duct ligation [3, 56]. However these effects remain controversial considering the results of contradictory studies [57, 58]. A human study has suggested an antifibrogenic effect induced by a synthetic agonist (pioglitazone) [59]. In HIV-infected patients with NAFLD, it has been shown that hepatic PPAR*γ* expression was significantly decreased compared to controls with normal liver histology and was inversely related to fibrosis [24]. In light of those results, PPAR*γ* agonists appear to be compelling drugs for the prevention of liver injury related to insulin resistance. PPAR*α* could be involved in fibrogenesis through adiponectin activation, as this adipokine could have antifibrogenic properties. Adiponectin has been shown to enhance PPAR*α* activation through the AMP-kinase pathway in animal models of cardiac fibrosis [60]. In HIV patients, a hepatic decrease of PPAR*γ* mRNA has been shown to be related to the presence and the severity of liver fibrosis [24].

## 3. Interactions between HIV and PPAR

### 3.1. Effects of HIV Infection on PPAR Activity

 The possibility that HIV-1 infection could influence PPAR*γ* activity has been established through in vitro studies.

In a recent study, HIV-1 viral protein r (Vpr) was shown to suppress the transcriptional activity of PPAR*γ* in mouse adipocytes. HIV could directly alter insulin sensitivity by suppressing PPAR*γ* activity [61]. The HIV Nef protein involved in viral replication has been shown to suppress PPAR*γ* expression [62].

### 3.2. Effects of PPAR on HIV

The ability of nuclear receptors to interact with the long-terminal of HIV-1 was recognized several years ago [63]. RXR and PPAR*α* were seen to bind a region between −356 to −320 in the long terminal repeat. In addition, PPAR*α* agonists such as clofibrate have been shown to activate HIV-1 transcription [64]. Fenofibrate, another PPAR*α* agonist also has properties that inhibit HIV replication and TNF*α* production in alveolar macrophages of HIV-infected patients [65].

In HIV-infected macrophages, natural and synthetic PPAR*γ* agonists inhibit HIV replication [60, 66]. More recently, it has been reported that HIV-1 replication was inhibited by ciglitazone, a PPAR*γ* agonist, in a dose-dependent manner in acutely infected human monocyte-derived macrophages and in latently infected and viral entry-independent U1 cells, suggesting an effect at the level of HIV-1 gene expression [66]. Cotransfection of PPAR*γ* wild-type vectors and treatment with PPAR*γ* agonists inhibited HIV-1 promoter activity in U937 cells, and activation of PPAR*γ* also decreased HIV-1 mRNA stability following actinomycin D treatment [67]. Similar results were observed by Skolnik et al. In this study, PPAR*α* and PPAR*γ* agonists decreased HIV-1 replication in peripheral blood monocular cells infected with HIV-1, in chronically infected monoblastoid cells and in alveolar macrophages from HIV-1 patients and controls [65]. The mechanisms of action by which PPAR acts on HIV-1 remain unknown. A direct effect on specific regions on HIV-1 and indirect effects via NF-*κ*B has been proposed [68].

### 3.3. PPAR Agonists as a New Therapeutic Path

Management of liver damage in HIV-infected patients requires increasing attention in regard to the growing liver-related mortality in these patients. The cofactors that are likely to worsen liver injuries need to be eradicated when possible: viral infection, alcohol, drug toxicity, overweight, and metabolic abnormalities. Lipodystrophy syndrome associated with insulin resistance plays a role in the development of fatty liver disease. By enhancing insulin sensitivity, PPAR*γ* agonists or thiazolinediones are used for the treatment of type 2 diabetes. In addition, benefits of these drugs have been suggested from experimental data conducted in vitro with PI-treated adipocytes [69]. As a consequence, several studies have been performed to evaluate the benefits of thiazolinediones in lipodystrophic HIV-infected patients. A recent study has evaluated the benefits of a treatment with pioglitazone (30 mg/d) compared to placebo among 130 HIV-infected patients with lipoatrophy. There was an increased amount of limb fat, but no significant difference in visceral abdominal fat, and the lipid profile (increased high-density lipoprotein) was improved [70]. Recent studies have suggested positive effects of thiazolinediones on steatosis and possibly fibrosis in NAFLD patients without HIV infection [59, 71]. Assessment of the benefits of pioglitazone on insulin resistance and liver injuries in HCV-infected patients is in progress (http://clinicaltrials.gov/show/NCT001891633). The main obstacle to using thiazolinediones is their cardiovascular side-effects, particularly in patients with increased cardiovascular risks, such as the HIV-infected patients treated with HAART. Such benefits need to be assessed in monoinfected and coinfected HIV-infected patients with fatty liver disease.

Fibrates are synthetic ligands of PPAR*α*, and they have been used for years in the treatment of lipid disorders. In HIV-infected patients, fibrates are efficient and safe in diet-resistant hyperlipidemia [72]. In a small cohort of uninfected patients with NAFLD, fenofibrate has been demonstrated to improve metabolic syndrome and liver tests without significant effects on liver histology [73].

The recently discovered endocannabinoid system contributes to the physiological regulation of food intake and glucose and lipid balance and is overactive in obese subjects. Two types of receptors have been described, CB1 and CB2, and are expressed in numerous tissues including the liver. An antagonist of CB1, called rimonabant, has been shown to induce weight loss and improve metabolic disorders in animals and humans [74]. In addition, this treatment could have antifibrogenic effects [75]. Interactions between the endocannabinoid system and PPAR*γ* ligands have been established, opening a new path for the management of metabolic-related liver injuries [76].

## 4. Conclusions

Liver diseases in HIV-infected patients both with and without viral hepatitis coinfection have received increasing attention in recent years. Metabolic disorders including insulin resistance, lipodystrophy, and NAFLD are long-term side effects that are frequently observed in HIV-infected patients receiving HAART.

Since the discovery of PPAR in 1990, significant progress has been made in understanding their effects and their potential roles in human disease and in metabolism alterations in particular. Animal and human experimental data have provided strong evidence for establishing a pathophysiological link between PPAR and NAFLD. Although the molecular mechanisms remain unclearly defined, direct reciprocal interactions between the virus itself and PPAR reinforce the hypothesis for the role of these transcription factors in the control of liver injury, particularly in steatosis, inflammation, and fibrosis. The existence of natural and synthetic ligands of PPAR opens new therapeutic options for the management of metabolic disturbances in HIV-infected patients with HAART-associated lipodystrophy, often associated with liver diseases. 
